# Supplementary figures and images for: Correlation between acoustic divergence and phylogenetic distance in soniferous European gobiids (Gobiidae; Gobius lineage)
Source: PLoS One. 2021 Dec 10;16(12):e0260810. doi: 10.1371/journal.pone.0260810 (PMC8664166; doi:10.1371/journal.pone.0260810)

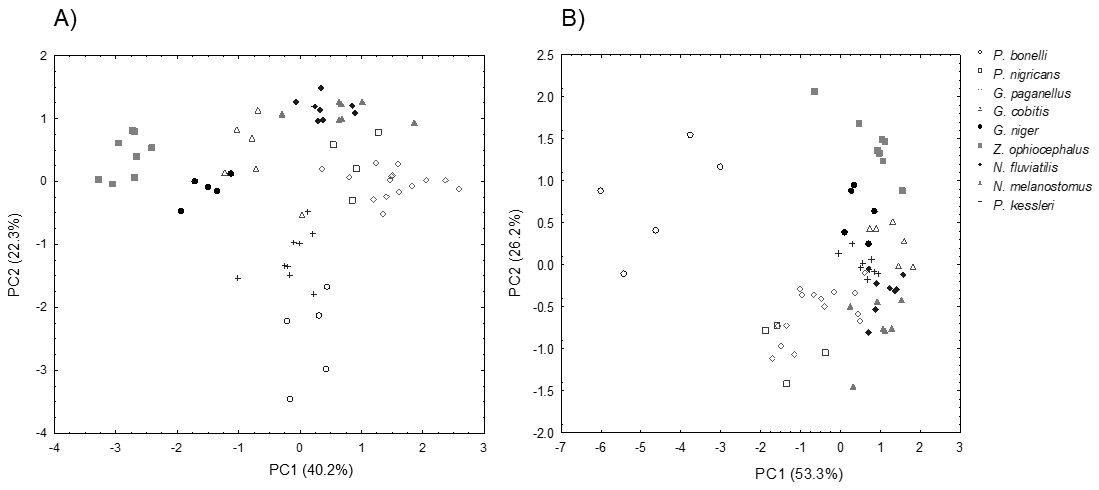

Supplement: S1 Fig — Scatterplot from principle component analysis (PCA) performed with individuals means of the five acoustic variables from nine gobiid species (Gobius lineage), performed A) without the correction on acoustic variable for size and B) with correction for size (“XTL-1”, where “X” is the acoustic variable). In A) PC1 is loaded by variables frequency modulation, pulse repetition rate and peak frequency, while PC2 by duration. In B) PC1 is loaded with pulse repetition rate, duration and sound rate, while PC2 by frequency modulation and peak frequency. (TIF) [file pone.0260810.s001.tif]

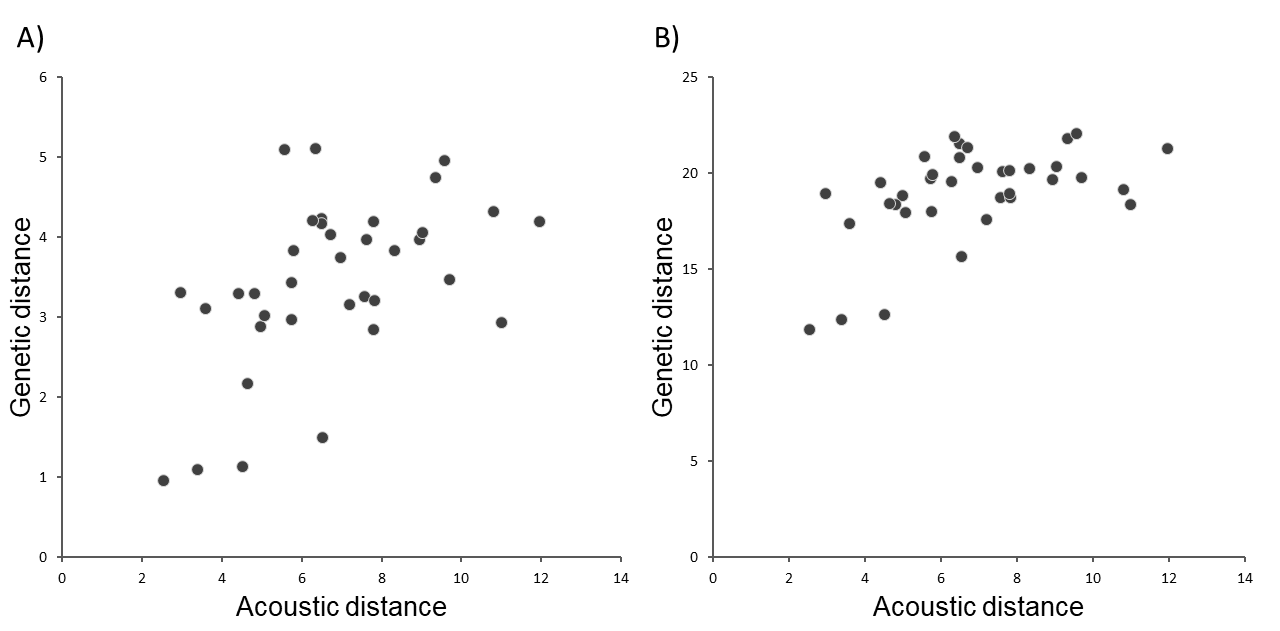

Supplement: S2 Fig — In A), correlation was achieved (Mantel test r = 0.48, Pt.t. = 0.005) by using genetic distance obtained from p-distance method for nuclear markers (rag1 and rho), while in B), correlation (Mantel test r = 0.45, Pt.t. = 0.01) was inferred from mitochondrial cytb and cox1 sequences while the divergence was obtained using p-distance method. The scatterplot represents the relationship between species genetic differentiation and their acoustic distance. (TIF) [file pone.0260810.s002.tif]
