# Supplementary material for: Correlation between acoustic divergence and phylogenetic distance in soniferous European gobiids (Gobiidae; Gobius lineage)
Source: PLoS One. 2021 Dec 10;16(12):e0260810. doi: 10.1371/journal.pone.0260810 (PMC8664166; doi:10.1371/journal.pone.0260810)
Supplement: S1 Table — For Perccottus glenii, only thump sounds were used for the acoustic analysis, for which FM could not be calculated (/). Number of recorded individuals per species (N) and number of analysed sounds (n) are indicated. Abbreviations: TL—total length, SR—sound rate, DUR—duration, NP—number of pulses, PRR—pulse repetition rate, PF—peak frequency, FM—frequency modulation. (PDF) [file pone.0260810.s003.pdf]

Table S1. Mean values and standard deviations of the total length and the six acoustic variables for the ten gobioid species. For *Perccottus glenii*, only thump sounds were used for the acoustic analysis, for which FM could not be calculated (/). Number of recorded individuals per species (N) and number of analysed sounds (n) are indicated. Abbreviations: TL - total length, SR - sound rate, DUR - duration, NP - number of pulses, PRR - pulse repetition rate, PF - peak frequency, FM - frequency modulation.

**Species (N)**	**n**	**TL (mm)**	**SR (s/min)**	**DUR (ms)**	**NP**	**PRR (Hz)**	**PF (Hz)**	**FM (Hz)**
P. bonelli (5)	50	75.6±7.9	18.2±6.6	679.2±145.7	57.6±5.6	90.6±7.9	134.2±11.4	-5.3±2.7
P. nigricans (4)	40	94.7±10.2	24.0±3.7	260.6±66.5	19.9±7.4	76.1±13.2	89.4±15.0	1.8±2.8
G. paganellus (15)	60	117.2±23.0	16.3±7.6	340.5±70.5	29.9±3.0	90.0±10.4	96.8±12.0	20.5±8.8
G. cobitis (6)	60	147.5±37.4	3.3±1.2	330.3±97.3	16.5±7.8	48.6±8.8	86.4±12.1	-2.5±7.3
G. niger (5)	30	121.4±12.4	8.4±4.3	368.2±32.6	16.4±2.5	44.7±5.9	109.4±11.2	-19.1±5.8
Z. ophiocephalus (8)	40	175.7±30.8	6.3±2.6	253.9±66.2	9.2±1.8	36.9±3.1	215.8±11.0	-13.6±3.3
N. fluviatilis (7)	80	130.6±10.8	4.4±5.5	170.0±39.5	12.4±2.5	73.7±6.9	78.0±5.8	8.8±4.7
N. melanostomus (7)	56	145.5±12.5	13.4±11.7	128.2±35.6	10.0±1.6	83.5±17.9	83.3±18.8	3.8±6.9
P. kessleri (9)	90	157.6±13.2	2.5±0.9	457.9±68.3	44.9±7.1	99.9±6.6	104.9±11.6	-9.1±10.6
P. glenii (6)	80	106.1±5.4	9.2±3.9	95.4±10.4	8.7±1.2	92.4±7.8	97.9±10.0	/
