# Supplementary material for: Correlation between acoustic divergence and phylogenetic distance in soniferous European gobiids (Gobiidae; Gobius lineage)
Source: PLoS One. 2021 Dec 10;16(12):e0260810. doi: 10.1371/journal.pone.0260810 (PMC8664166; doi:10.1371/journal.pone.0260810)
Supplement: S2 Table — (PDF) [file pone.0260810.s004.pdf]

Table S2. Partitioning scheme and best-fit models of evolution for data blocks defined by gene and codon position, assessed by PartitionFinder 2 for subsequent phylogenetic analyses in MrBayes and RAxML.

**Best-fit model**	**MrBayes settings for assessed models**	**Best-fit model for RAxML**	**Partitioning scheme (gene-codon position)**
HKY+I	nst=2, rates=propinv	GTR+G	*cox1*-3, *rho*-1, *cytb*-1
TRN+G	nst=6, rates=gamma	GTR+G	*cytb*-2, *cox1*-1
K80+G	nst=2, rates=gamma, statefreqpr=fixed	GTR+G	*cytb*-3
TRNEF+G	nst=6, rates=gamma	GTR+G	*cox1*-2
K81UF+I	nst=6, rates=propinv	GTR+G	*rag1*-1, *rho*-3
K80+G	nst=2, rates=gamma, statefreqpr=fixed	GTR+G	*rag1*-2
TVM	nst=6	GTR+G	*rho*-2, *rag1*-3
