# Supplementary material for: Correlation between acoustic divergence and phylogenetic distance in soniferous European gobiids (Gobiidae; Gobius lineage)
Source: PLoS One. 2021 Dec 10;16(12):e0260810. doi: 10.1371/journal.pone.0260810 (PMC8664166; doi:10.1371/journal.pone.0260810)
Supplement: S3 Table — Correlation is based on the individual means of six acoustic properties per species (N = 9). (PDF) [file pone.0260810.s005.pdf]

Table S3. *Spearman* correlation coefficient of the relationships between the six acoustic properties. Correlation is based on the individual means of six acoustic properties per species (N = 9).

Variable	SR	DUR (ms)	NP	PRR (Hz)	PF (Hz)	FM (Hz)
SR (s/min)	1.000	0.037	0.102	0.035	0.050	0.318
DUR (ms)	0.037	1.000	0.886*	-0.006	0.178	-0.201
NP	0.102	0.886*	1.000	0.360	0.052	0.109
PRR (Hz)	0.035	-0.006	0.360	1.000	0.115	0.326
PF (Hz)	0.050	0.178	0.052	0.115	1.000	-0.435*
FM (Hz)	0.318	-0.201	0.109	0.326	-0.435*	1.000

Asterisk (*) denotes a significant correlation less than $P < 0.001$.
