# Supplementary material for: Correlation between acoustic divergence and phylogenetic distance in soniferous European gobiids (Gobiidae; Gobius lineage)
Source: PLoS One. 2021 Dec 10;16(12):e0260810. doi: 10.1371/journal.pone.0260810 (PMC8664166; doi:10.1371/journal.pone.0260810)
Supplement: S4 Table — PC factor coordinates represent the correlations between the respective individual mean value of sound variable and each PC factor. PC (without) indicate the percentages and loadings of acoustic variables obtained without the correction for size, while PC (with) highlights the percentages and loadings obtained with the acoustic variables corrected for size (“XTL-1”, where “X” is the acoustic variable). For PCAs, we excluded acoustic variable number of pulses (NP) due to its correlation with other variable (DUR). (PDF) [file pone.0260810.s006.pdf]

Table S4. Percentage and cumulative percentage of variance explained by the first two axis of principal component analysis (PCA), with the loadings for these axes (i.e., factor coordinates) extracted from five acoustic variables from nine gobiid species (*Gobius* lineage). PC factor coordinates represent the correlations between the respective individual mean value of sound variable and each PC factor. PC (without) indicate the percentages and loadings of acoustic variables achieved without the correction for size, while PC (with) highlights the percentages and loadings achieved with the acoustic variables corrected for size ("XTL^{-1}", where "X" is the acoustic variable). For PCAs, we excluded acoustic variable number of pulses (NP) due to its correlation with other variable.

Variable	PC1 (without)	PC2 (without)	PC1 (with)	PC2 (with)
Percentage	40.20	22.38	53.31	26.28
Cumulative percentage	40.20	62.58	53.30	79.58
SR (s/min)	0.54	-0.07	-0.81	-0.24
DUR (ms)	0.00	-0.95	-0.82	0.25
PRR (Hz)	0.73	-0.35	-0.85	-0.27
PF (Hz)	-0.71	-0.14	-0.74	0.48
FM (Hz)	0.82	0.23	-0.14	-0.93
