# Supplementary material for: Correlation between acoustic divergence and phylogenetic distance in soniferous European gobiids (Gobiidae; Gobius lineage)
Source: PLoS One. 2021 Dec 10;16(12):e0260810. doi: 10.1371/journal.pone.0260810 (PMC8664166; doi:10.1371/journal.pone.0260810)
Supplement: Attachment — Submitted filename: Response to Reviewers.docx [file pone.0260810.s009.docx]

**REBUTTAL LETTER**

- **Reviewer #2: The present version of the manuscript PONE-D-21-00246R1 from Horvatić et al., has been revised and the authors conformed with most comments and suggestions. Editions were properly made where authors agreed with the comments, and explanations were provided when the authors’ choice was to not comply with the suggestions. The overall writing is clearer, allowing an intelligible reading flow. Some of the suggested analyses were done, figures were modified, clarifying interpretations. The authors wisely moderated the assumptions and reshaped a more focused discussion leading now to an adequate philosophy of the general conclusion. However, a few remaining points suggested below would be critical to address and consider.**

We are satisfied that our resubmitted manuscript, after provided Major revision, follows the suggestions proposed by both reviewer(s). In addition, we are aware that some of the minor errors were still present in this resubmitted version, and in this sense, we gave our best to fulfil the demands of the reviewer #2 in order to strengthen our hypotheses and produce high quality manuscript within the current revision process. Therefore, we believe that this version of the manuscript will meet the standards and prerequisites for its acceptance in world’s leading scientific journals such as *Plos One*.

**MATERIAL AND METHODS SECTION**

- **Line 132. Please write slightly more explicitly e.g. “availability of previously recorded audio tracks…”**

We agree, therefore, we have corrected this part as you suggested.

- **Line 153. Replace “life-history” trait by “morphological trait”**

We agree, and according to this suggestion, we have corrected this part.

- **Line 155. Table S1 and Table S2 should be inverted and appear according to the chronology of the reading. Please modify where it applies in the manuscript and supplementary accordingly.**

We completely agree, and this was corrected accordingly throughout the manuscript.

**RESULTS SECTION**

- **Line 349. The PF (Hz) also looks associated with PC1 with a score of -0.71. Please, comment unless the value is under an arbitrary threshold set by the authors which should, in that case, be mentioned somewhere, here or in the material and method section.**

The value was not under the arbitrary threshold, but was simply unrecognized or neglected by the authors during the initial writing period. Therefore, we have included the *PF* into the account when discussing the results of PCA analysis.

- **Line 353. “PCA accounted cumulatively for 80% of variation” Where is this shown, could the authors add that to Table S3 and precise which PCAs were done with or without TL correction.**

We agree with your comment, and following your suggestions, we have decided to improve *Fig. S1* and *Table S3* by including the information from two PCA (one *without* and one *with* TL correction).

- **Phylogenetic affinities between soniferous gobiids: the morphological parameters i.e. swim bladder and numbers of vertebrae should not be disregard while the authors describe the tree in Fig5, in the manner of the environment is quickly commented.**

We agree, therefore, we have included additional textual explanations within the revised manuscript in order to cover your suggestions regarding morphology (*n. of vertebrae and swim bladder*) and to facilitate the reading/understanding of the *Fig. 5*.

- **Line 441-447. Overall, please, rewrite that paragraph a little clearer to facilitate the reading i.e. avoid back and forth, repetitions or cross-descriptions of the tree.**

We agree, therefore, we have revised and rewrote this section according to your suggestions.

- **Line 442. A little confusing “Two marine gobies…” Please rephrase to clarify that four of marine gobies are spread into two distinct clades, e.g. one clade with Gobius niger and Zosterisessor ophiocephalus and another one including G. cobitis and G. paganellus.**

We agree, and we have corrected this part of the manuscript. Now, the sentence formulation goes: “*From four marine gobies (which share the reduced number of vertebrae [< 28] and the presence of swim bladder), Gobius niger and Zosterisessor ophiocephalus grouped into one clade, while G. cobitis and G. paganellus clustered into the second.*”

- **Line 445-447. Confusing, please rephrase clearer “In the third group, P. kessleri […] in regard to these two species.”**

We agree, therefore, the mentioned section was improved according to your suggestions: “*The third group was composed of gobiids distributed in the Ponto-Caspian region (genera Neogobius and Ponticola) and P. nigricans, an Italian freshwater endemic species (Fig 5). This group occupies freshwater habitats, has and elevated number of vertebrae (> 28) and lacks a swim bladder. Specifically, in the third group, P. kessleri is a sister taxon to the remaining species, while within the “Neogobius group”, Neogobius fluviatilis is a sister taxon in regards to two closely related species, P. nigricans and N. melanostomus”.*

**DISCUSSION SECTION**

- **Line 528. Please, add as suggested to remove any ambiguity here as well “…according to their observed frequency in the behavioural experiments the sounds were extracted from.”**

We agree, therefore, this part was corrected as suggested.

- **Line 550. Change “this” by “our”, or by “the present study” for precision, e.g. “…corroborated by the present study”.**

We agree, therefore, this part was corrected as suggested.

- **Line 552-553. “This is in agreement with…” The statement does not argue in favour of the findings of the present study showing that P. bonelli ranks separately from the other species described line 550-552. The authors focused on the most represented sound within species, including in P. bonelli that is able to produce other sound types. Since the other sound types were not investigated for that species here, it is not possible to ascertain that acoustic clusters in PCA, DFA or else would have been similar. Please, remove or replace by another argument to discuss that P. bonelli ranks separately.**

We agree, therefore, we have eliminated this sentence from the revised manuscript.

**FIGURE LEGENDS SECTION**

- **Line 367. Fig2. Explain somewhere (in the figure caption for example) what letters correspond to, i.e. significant differences between which species.**

If we understood correctly this comment, the proposed suggestion is to explain each letter for each species within the Fig 2. If this is the case, we believe that including the explanation for each letter would take too much space in the figure caption, or especially, in the text. In addition, by including the letter explanations, the graphs within the Fig 2. become unnecessary and the Figure redundant. From the present figure caption and Fig 2. itself, we believe it should be simple to conclude that species with letter *a*, *b*, *c*, etc. are significantly different, and that the ones with the combinations of letters (*bc*, *adc*, etc.) share similar properties. To further strengthen our rationale for not including the explanations for each letters in text/caption, we propose two (of many) papers where the similar approach has been followed (Velasquez et al., 2013 “*Bioacoustic and genetic divergence in a frog with a wide geographical distribution*” & Lee et al., 2016 “*Geographic variation in advertisement calls of a Microhylid frog – testing the role of drift and ecology*”). Therefore, we strongly believe there is no need for the inclusion of the explanations of the different letters, especially considering the Fig 2. which would in that case become redundant.

- **Line 414. Fig4. Please annotate in that legend what PRR and NP stand for.**

We agree, therefore, we have included explanation (*NP - number of pulses, PRR - pulse repetition rate*).

- **Line 458. Fig5. Explain scale bar 0.2, i.e. one should easily read whether/how the branches are time-scaled.**

We agree, therefore, we have included the explanation in the figure caption: “*Branch length scale represents number of substitutions per site*.”

- **Line 476. Table 5. Explain the empty box column “1.” for *G. cobitis*.**

After initial revision, the idea was to highlight the correlation between different species within the Table 5, while giving the dash (-) to the states where we have noticed intercorrelation within the same species (e.g. *G. cobitis* vs *G. cobitis*). Afterwards, we have noticed that the practical solution for genetic distance matrices (as proposed by the MEGA software instructions and as noted in many phylogenetic papers, such as Buj et al., 2017 “*Ancient connections among the European rivers and watersheds revealed from the evolutionary history of the genus Telestes (Actinopterygii; Cypriniformes)*” or Grzywacz et al., 2017 “*Evolution and systematics of Green Bush-crickets (Orthoptera: Tettigoniidae: Tettigonia) in the Western Palaearctic: testing concordance between molecular, acoustic, and morphological data*”) is to leave the empty cell with the intercorrelation coefficient within the same species (e.g. *G. cobitis* vs *G. cobitis*). We have followed the same approach in this revised manuscript, and therefore, we eliminated the dash from *Table 5* while simply leaving the *cell empty* for *G. cobitis* vs *G. cobitis*, *G. paganellus* vs *G. paganellus*, etc...

**FIGURES**

- **Fig3. Double-check for the accuracy of the numbers of plotted individuals in the DFA, e.g. number of empty triangles showing G. cobitis.**

We have checked and solved the suggested problem. The “real” number of *G. cobitis* individuals was always 6, so the initial confusion was produced probably due to the Table S1, where we unintentionally indicated that we have used 4 individuals of *G. cobitis*. This was corrected in the revised manuscript (in *Table S1* we have corrected 4 into *6* *individuals*). If you count the triangles in PCA and DFA, there are really six of them, meaning that we have used *six individuals* of *G. cobitis* for our acoustic analyses.

- **Fig6. Please, increase the size of the legend which is currently difficult to read; similarly, with the name of the species.**

We agree, and therefore, we have corrected this part as suggested.

**TABLES**

- **Line 370. Table 2. Could be placed in Suppl data e.g. with Table S3 to facilitate the reading, since the main correlated parameters already appear in the main text. Clarify whether this correlation considers the TL correction.**

We agree, therefore, we have moved *Table 2*. into *Supplementary data*, so in the revised manuscript, this table is referred to as *Table S3*.

- **Suggestion to exchange Table S1 and table S2, the latest is commented earlier in the manuscript.**

This was corrected earlier.

- **Table S2. Briefly explain the empty FM box (Hz) for P. glenii somewhere. Double-check that the number of recorded individuals set in Table S2 matches with the number of individuals shown in all analysis, tables and plots and everywhere else in the manuscripts, e.g. G. cobitis.**

This was corrected earlier.

- **Table S3. Please check the legend for the accurate number of acoustic variables included in the present PCA and quickly mention the rational for removing NP here. Mention here or somewhere else appropriate when PCAs were done with or without TL correction.**

We agree, therefore, we have included the rationale for removing NP within the *Table S4* (“*For PCAs, we excluded acoustic variable number of pulses (NP) due to its correlation with other variable (DUR)”*), and corrected *six* into *five* *variables*. The TL corrections was explained and corrected earlier.
